# Psychological Climacteric Symptoms and Attitudes toward Menopause among Emirati Women

**DOI:** 10.3390/ijerph17145028

**Published:** 2020-07-13

**Authors:** Amira Mohammed Ali, Afaf Hassan Ahmed, Linda Smail

**Affiliations:** 1 Department of Mental Disorder Research, National Institute of Neuroscience, National Center of Neurology and Psychiatry, Tokyo 187-0031, Japan; 2 Department of Psychiatric Nursing and Mental Health, Faculty of Nursing, Alexandria University, Alexandria 21527, Egypt; 3 Department of Obstetrics and Gynecologic Nursing, Faculty of Nursing, Alexandria University, Alexandria 21527, Egypt; drafafhassan445@gmail.com; 4 Department of Mathematics & Statistics, Zayed University, Dubai 19282, UAE; linda.smail@zu.ac.ae

**Keywords:** fatigue, depression, anxiety, sleep, memory, psychological distress, vasomotor symptoms, sexual symptoms, body weight/obesity, menopause, quality of life, attitudes toward menopause, Arab, Emirate

## Abstract

Menopause is an inevitable developmental event that women encounter at an age of 42–54 years. The drop of estrogen levels that accompanies cessation of menstruation is associated with multiple vasomotor, physical, neuropsychological, and sexual symptoms, which may hamper quality of life. This study aimed to examine the severity of psychological symptoms and their correlates among peri- and postmenopausal Emirati women (N = 60, mean age = 54.88 ± 6 years). Participants were interviewed using the Menopause-Specific Quality of Life (MENQOL) and attitudes toward menopause scale (ATMS). In four path analysis models, vasomotor symptoms, weight gain, and fatigue had significant direct effects on symptoms of anxiety, depression (only weight gain and fatigue), and psychological distress. Fatigue significantly mediated the effects of vasomotor symptoms and weight gain on symptoms of anxiety, depression (only vasomotor symptoms), psychological distress, and memory problems. These models explained 47.6%, 44.5%, 56.6%, and 29.1% of the variances in anxiety, depression, psychological distress, and memory problems, respectively. Participants with more severe menopausal symptoms expressed more negative attitudes toward menopause though regression analysis revealed that only vasomotor symptoms could significantly contribute to ATMS scores. In conclusion, psychological distress is widespread among menopausal women, and it is associated with vasomotor symptoms, fatigue, and change of body composition (obesity). Psychological symptoms, along with vasomotor symptoms, express a key link to negative attitudes toward menopause. Therefore, interventional strategies that target psychological distress may promote coping with midlife transition and improve mental health among menopausal women.

## 1. Introduction

Menopause transition is characterized by a massive drop of estrogen levels (the main feminine sex hormone), ovarian failure, and menstrual irregularities [1]. Menopause is described as cessation of menses for 12 sequential months after the last period [2]. It is a universal physiological condition that annually affects more than 500 million women aged 42 to 55 years with an average age of onset of 51 years [2,3,4]. Hormonal changes that accompany the onset of menopause trigger the development of several physical, sexual, vasomotor, and psychological symptoms [4].

Compared with premenopausal women, menopausal women express a wide range of psychological symptoms including poor memory and concentration, depression, anxiety, insomnia, fatigue, irritability, and a high level of distress, which may impede coping and decrease quality of life in this group [5]. Furthermore, severe neuropsychiatric symptoms may reflect the development of age-related pathologies such as cognitive impairment associated with Alzheimer’s disease, especially during the prodromal stage, but get misinterpreted as symptoms of natural aging and go uncared for [6], which can have serious health-related drawbacks. 

The Arab world extends on an area of 5 million square miles in 2 major continents, Asia and Africa. It comprises 22 countries inhabited by 423 million people who share the same language, as well as the same cultural, historical, and religious heritage [7,8]. Although several studies assessed menopausal symptoms in women from different Arab countries, vasomotor and physical symptoms have been the focus of most studies [4,9,10] while less attention has been paid to neuropsychiatric symptoms. In this light, the current study attempts to fill this gap by exploring the nature of common menopausal psychological symptoms among Emirati menopausal women as a sample of Arab women.

Forgetfulness and reductions in attention, processing speed, and verbal fluency (indicated by difficulty finding words) are common cognitive problems endorsed by menopausal women and women in the menopause transition [11,12]. Declines in memory represent the second most frequent menopausal symptom after vasomotor symptoms and joint stiffness, and the severity of these symptoms can be quite alarming [1,12]. Estrogen deficiency alters brain structure and function, resulting in symptoms of cognitive aging and susceptibility to Alzheimer’s disease in genetically vulnerable individuals [11]. In particular, estrogen, a neurosteroid with multiple neuroprotective effects, interacts with brain cells through its widely distributed estrogen receptors (ERs) β and α to regulate key processes relevant to executive functioning and memory: signal transduction and neurotransmission (e.g., of acetylcholine, serotonin, noradrenaline, and glutamate) in the prefrontal cortex, synaptic plasticity, production of neurotrophic factors such as nerve growth factor, neurite growth, dentate gyrus neurogenesis, DNA repair, mitochondrial production of adenosine triphosphate, and production of internal antioxidants [11,13,14,15]. 

Symptoms of depression, anxiety, and sleep disturbance are other neuropsychiatric symptoms linked to cognitive performance at midlife transition in women, but they do not explain memory declines in menopause [12]. Women are at a greater risk for depression than men, and such risk heightens with aging [16]. Women in menopausal transition are at two- to fourfold higher risk for major depressive disorders than premenopausal women [17]. Perimenopausal women often experience different depressive symptoms (e.g., low mood, lack of motivation, lack of pleasure sense, and disrupted sleep), which can severely impair their quality of life [17]. A recent large-scale cross-sectional study reported a diagnosis of depression (on the Center for Epidemiologic Studies Short Depression Scale-10) among 18.4% of community-dwelling middle-aged women in Canada. Women experiencing menopause before the age of 40 endorsed the highest incidence of depression (odds ratio = 1.45; CI: 1.07–1.97) [18]. Likewise, surveying Turkish menopausal women with Beck depression inventory (BDI) revealed that 27.5% of them are affected by depression [19].

Menopause-related estrogen decline may trigger mood dysregulation by impeding the production of major neuroprotective factors (e.g., brain-derived neurotrophic factor) and, by altering neurotransmission, it interferes with the synthesis of catecholamines such as noradrenaline and upregulates 5-hydroxytryptamine (serotonin) (5-HT2A) receptor [1,11]. The latter contributes to hot flashes by modulating the set point temperature [1]. Vasomotor symptoms (e.g., hot flashes and night sweats), the cardinal symptoms of menopause, are experienced by 85% of perimenopausal women [1,12]. These symptoms are quite bothersome, and they represent a major risk factor for sleep disturbances as well as for anxiety and/or depressive symptoms [16,18]. A current study comprising a sample of 13,216 Canadian women aged 45 to 64 years reports increased severity of vasomotor symptoms and higher odds of depression (odds ratio = 1.21; CI: 1.02–1.44) among postmenopausal women on hormonal replacement therapy (HRT, also known as menopause hormone treatment) [18]. Mood changes that affect menopausal women are associated with numerous factors other than hormonal declines such as genitourinary symptoms (e.g., vaginal dryness/dyspareunia), less education, nonwhite ethnicities, history of smoking, history of anxiety and postpartum depression, nulliparity, obesity, stressful life events, poor social support and partner death, low self-esteem, unemployment, encountering multiple stressors, and poor coping style [11,16,18,20].

Advanced age is associated with disturbed sleep (insomnia, nighttime awakening, or waking early) both in men and women. However, sleep loss in women is approximately twice that of men [1,12]. Both subjective self-reports and actigraphy studies uncover poor quality and quantity of night sleep among women during their late reproductive years, especially before the time of menses. Sleep problems that occur because of night sweat and hot flashes are tightly linked to hormonal declines [1]. Meanwhile, sleep problems in women aged 40 years and older are attributed to many factors other than estrogen deficiency such as having anxiety and/or depressive disorders, socio-economic difficulties, white race, and marital conflicts [1,11,21]. Daytime fatigue and sleepiness associated with inadequate sleep may contribute to injury, depressive symptoms, and poor quality of life [1]. 

Menopausal symptoms such as lack of energy and fatigue, experienced by 43.9–64.7% of menopausal women, are strongly associated with exhaustion and burnout, which may seriously compromise quality of life of affected women [22,23,24]. Fatigue is described as a feeling of lack of energy, weariness, loss of drive, decrease or loss of ability to sustain even routine activities, overwhelming feeling of tiredness, exhaustion, and physical or mental strain that occurs even without conspicuous effort. Pathological fatigue is an intensified level of the common “physiological” fatigue, and it may be evoked by pervasive pathologies [24,25,26]. The relation between fatigue and stress is dynamic, and it drastically changes during menopausal transition [22]. In fact, chronic fatigue in humans is described as a stress-related condition that involves numerous systemic dysfunctions, which alter homeostasis and hinder women’s ability to bounce back from either fatigue or distress [5,22]. For instance, dysregulation of the hypothalamic–pituitary–adrenal axis and persistent activation of the sympathetic nervous system are common in people with chronic fatigue syndrome, and they trigger a wide range of symptoms that can induce excessive suffering [5,26]. Studies on menopause-specific fatigue, fatigue explained mainly by endorsing menopause transition rather than suffering insomnia or depression, are relatively scarce [23]. However, evidence denotes strong connections between menopausal fatigue and emotional negativity (depressed mood and perceived stress) during menopause [27]. Fatigue in general is usually accompanied by several unpleasant symptoms such as pain, distress, insomnia, impatience, irritability, and mood disturbances [5,21,23,26,28]. In this regard, menopause-related fatigue is reported to be a key source of exhaustion, burnout, and low resilience among working nurses. Meanwhile, resources of social and organizational support could not mitigate its effects. Hence, working menopausal women may require specific interventions that promote processes of energy recovery [22]. Research shows that menopause-specific fatigue can be alleviated by pharmacologic treatments (e.g., armodafinil, a wakefulness-promoting agent and a central nervous system stimulant) and non-pharmacologic interventions such as hypnosis [23,29].

Weight status is a key effector of menopausal and mood symptoms in peri- and postmenopausal women [27,30]. Aging is associated with oxidative stress and chronic inflammation, which promote the buildup of saturated ceramide and diacylglycerol fatty acids resulting in increased total body fat and visceral adipose tissue [31,32]. Emotional distress associated with menopausal symptoms boosts the production of cytokines and free radicals resulting in more fat deposition/obesity [27]. On the other side, obesity contributes to the development of neurodegenerative disease such as depression, anxiety, and Alzheimer’s disease through a complex mechanism. On one hand, cerebral insulin resistance develops in obese individuals due to depletion of adiponectin—an adipose tissue-derived adipokine that regulates neurogenesis and the metabolism of lipids and glucose [33]. On the other hand, obesity involves pathological reshaping of gut microbiome structure and activation of major oxidative and inflammatory pathways, which fuel neuronal inflammation—an essential aspect in the development of mood and cognitive disorders [34,35]. Moreover, research shows that high fat mass interferes with the effect of therapeutic interventions used to manage menopausal symptoms such as physical exercise [27].

Attitudes toward menopause represent believes held by women about bodily and role changes that are associated with feminine aging. Some psychologists view psychological complaints expressed by menopausal women as reflections of cultural expectations [36]. In addition, research highlights associations between negative attitudes toward menopause and the severity of menopausal symptoms (e.g., vasomotor symptoms and fatigue) [10] and psychological symptoms [19]; however, findings are inconsistent. Attitudes toward menopause vary considerably between different cultures. They tend to be more positive when associated with positive changes in social status [19]. For example, menopausal women in Japan express more positive attitudes toward menopause compared with women in Western countries [36]. In this light, attitudes might threaten femininity in societies that consider it as a partial death of a woman [36]. Several factors affect the extent to which menopausal women develop positive or negative attitudes toward this life transition. An available systematic review reports that husbands’ perceptions of and attitudes toward menopause may largely influence marriage relationships, women’s menopausal symptoms, and attitudes toward menopause [37]. The term used to express menopause in Arabic is “sen al yaas”, which means “the age of despair or hopelessness”. Despite the negative connation associated with menopause in Arab countries, in depth investigations show that Arab postmenopausal women receive proper support from their husbands, participate in religious activities that they could not take part in before, and demonstrate more socially-active roles than before [2,38]. In addition, the term “age of despair or hopelessness” has been recently changed in the media by a more positive term “the age of wisdom or respect”. 

A former systematic review reports a great variability in the age of onset of menopause and the occurrence of vasomotor symptoms worldwide, with the lowest age occurring in Asia (42.1–49.5 years) compared with Europe and North America (50.1–52.8 years and 50.5–51.4 years, respectively) [39]. The United Arab Emirates (UAE) is a small country located in the Gulf region in Asia. It has witnessed remarkable improvements in the health system during the last few decades, which were associated with increased life expectancy (up to 83.4 years) [2,40]. The median age of menopause in the UAE is 48 years (mean age = 47.3 ± 3.29, range 40–59), which is significantly lower than the median age reported in Western countries (50.3 years) [40]. Given the remarkable increase of lifespan in the UAE, women are expected to endure postmenopausal symptoms and complications for around one third of their lives [2]. Although menopausal symptoms have been previously evaluated in the UAE, less attention has been paid to neuropsychiatric symptoms such as depression, anxiety, memory, and sleep problems [4]. Similarly, attitudes toward menopause have been studied in the UAE before [2]. However, the association between menopausal symptoms and attitudes toward menopause in the UAE has not been assessed yet. Therefore, the current study offers in depth exploration of psychological climacteric symptoms and addresses the relationship between menopausal symptoms and attitudes toward menopause. 

**Figure 1:**
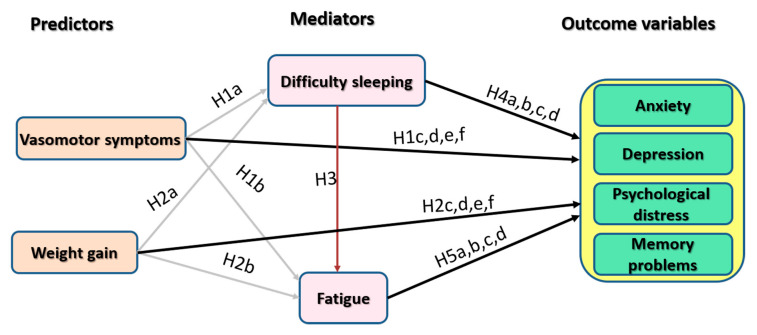
Hypothesized model of the relationships between vasomotor symptoms, weight gain, difficulty sleeping, fatigue, and psychological variables.

Based on the aforementioned background, this study aimed to examine the direct and indirect effects of vasomotor symptoms and weight gain on psychological symptoms of menopause. Specifically, we hypothesized that vasomotor symptoms and weight gain contribute to sleep loss/difficulty sleeping and fatigue, which in turn contribute to anxiety and depressive symptoms, psychological distress, and memory problems (Figure 1). We also examined the association between neuropsychiatric climacteric symptoms and other factors documented in the literature to contribute to distress, e.g., sexual symptoms, age, menopausal status (perimenopausal vs. postmenopausal), short time since menopause onset, level of education, smoking, employment, marital status, and HRT use [30,40]. The second aim of the current study was to identify correlates of attitudes toward menopause. In particular, we postulated that women experiencing more severe symptoms would endorse more negative attitudes toward menopause.

**Hypothesis 1a** **(H1a).** *Vasomotor symptoms contribute to difficulty sleeping.*

**Hypothesis 1b** **(H1b).** *Vasomotor symptoms contribute to fatigue.*

**Hypothesis 1c,d,e,f** **(H1c,d,e,f).** *Vasomotor symptoms contribute to anxiety, depression, psychological distress, and memory problems.*

**Hypothesis 2a** **(H2a).** *Weight gain contributes to difficulty sleeping.*

**Hypothesis 2b** **(H2b).** *Weight gain contributes to fatigue.*

**Hypothesis 2c,d,e,f** **(H2c,d,e,f).** *Weight gain contributes to anxiety, depression, psychological distress, and memory problems.*

**Hypothesis 3** **(H3).** *Difficulty sleeping contributes to fatigue.*

**Hypothesis 4a,b,c,d** **(H4a,b,c,d).** *Difficulty sleeping contributes to anxiety, depression, psychological distress, and memory problems.*

**Hypothesis 5a,b,c,d** **(H5a,b,c,d).** *Fatigue contributes to anxiety, depression, psychological distress, and memory problems.*

## 2. Materials and Methods

### 2.1. Study Design, Participants, and Procedure

This study is a secondary analysis based on data of a formerly published study [4]. In brief, a multistage random sample comprising 70 women aged 40–64 years was collected from two public health care centers in Dubai between April and August 2018. Data were collected through face-to-face interviews that were conducted by five trained nurses. The original sample included 10 premenopausal women, which were not included in the current analysis because the literature reports significant differences in neuropsychiatric symptoms between premenopausal women and their peri- and postmenopausal counterparts [5,17]. 

### 2.2. Study Instruments

Participants of this study were interviewed face-to-face using a structured questionnaire that consisted of three sections. The first section involved assessment of sociodemographic, clinical, and menopause-related data, e.g., age, education, smoking, health status, date of the last menses, HRT use, etc. The second section comprised the Menopause-Specific Quality of Life (MENQOL) scale developed by Hilditch et al. [41]. The MENQOL scale contains 29 items, which explore the experience of different menopausal symptoms as endorsed or not endorsed as well as the degree of being bothered by symptoms on a scale that ranges from 0 (not at all bothered) to 6 (extremely bothered). Symptoms assessed by the MENQOL are distinguished into four domains: vasomotor (items 1–3), psychosocial (items 4–10), physical (items 11–26), and sexual (items 27–29) [9,42]. The questionnaire was translated into Arabic by a professional translator. The Arabic version was checked by a bilingual expert who back translated it into English. Then, a panel comprising a professor and a consultant specialized in obstetrics and gynecology checked the content validity of the scale, which was then tested for reliability in a small-scale sample (15 women) by Smail and others [4]. Reliability of the MENQOL, as evaluated in our sample, is excellent (α = 0.96). The third section comprised the attitudes toward menopause scale (ATMS) developed by Neugarten et al. (reviewed in [2]). ATMS is a four-point Likert scale (1 strongly agree, 2 agree, 3 disagree, 4 strongly disagree), which consists of 34 items, which examine attitudes toward menopause in general. For each item, responses range from strongly agree to strongly disagree. Positive items are reverse coded. Accordingly, greater ATMS scores reflect positive attitudes and low scores reflect more negative attitudes toward menopause. Validity and reliability of ATMS are documented in the literature [2]. Reliability of ATMS, as evaluated in our sample, is acceptable (α = 0.79).

### 2.3. Ethical Considerations

The original study was approved by the Research and Ethics Committee at Zayed University and Dubai Health Authority Research Office. Permissions from the selected primary health care centers were obtained. Selected women were informed that data are collected for scientific purposes only, and they were invited to participate in the study on a voluntary basis. A consent form was given to each participant, and they were assured about the confidentiality, anonymity, and security of data. To protect the confidentiality of the participants, data collected were stored in a locked file cabinet in a supervised office during the study. After the end of data collection, all identifying personal information were destroyed.

### 2.4. Statistical Analysis

**Figure 2:**
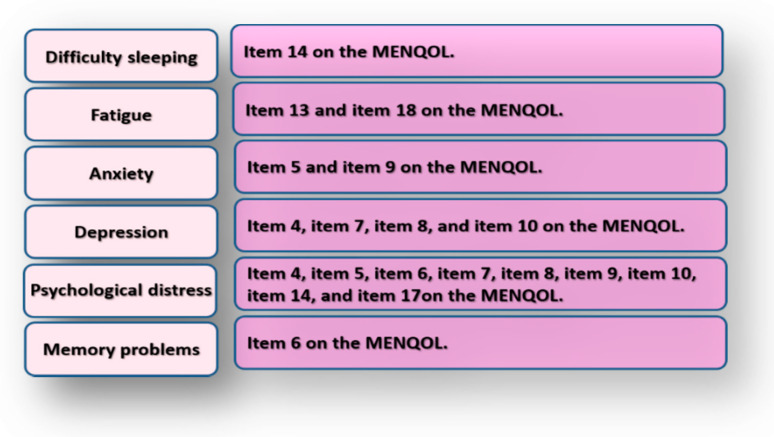
Definitions of key variables in this study. MENQOL: Menopause-Specific Quality of Life scale.

For analysis, we converted item scores of the MENQOL scale, which represent subject responses, into a score ranging from 1 (not experiencing a symptom) to 8 (extremely bothered by the symptom). The literature indicates that item scores of the psychosocial domain of the MENQOL correlate with measures of depression and psychological health such as BDI and the Short Form-36 questionnaire. In addition, BDI scores correlate with overall scores of the MENQOL scale [42]. Therefore, we examined the scale for other items related to psychological health. We found that the physical domain contains several items that express psychological problems: items 13 “feeling tired and worn out”, 14 “difficulty sleeping”, 17 “decrease in stamina”, and 18 “feeling lack of energy”. Similar items are included in the Depression Anxiety Stress Scale—a measure of depression, anxiety, and psychological stress [43,44,45]. Besides, insomnia and fatigue “e.g., feeling tired or lacking energy” are reported to be psychological symptoms commonly experienced by menopausal women [5,22,27]. Accordingly, we conducted exploratory factor analysis with Oblimen rotation, and it revealed that some items of the physical domain cross-loaded on other domains (Supplementary Materials). Hence, we reconstructed the psychosocial domain in the current analysis to comprise 2 extra items by adding item 14 and item 17. Internal consistency reliability of our psychosocial domain of the MENQOL is excellent (α = 0.90). Item 13 and item 18 were combined to calculate fatigue scores. Definitions of all psychological variables addressed in this study are illustrated by Figure 2. 

Responses to items of the ATMS range from 1 to 4, and ATMS data were treated as numerical and managed on the interval scale according to Harpe (2015) [2,46]. Accordingly, we created a variable named the “average attitudes score” by computing the average of each participant’s responses to all items of the ATMS. This variable was used to represent ATMS in the analysis.

We reported the percentage of participants encountering psychological symptoms and the percentage of participants endorsing attitudes investigated by ATMS as well as median and interquartile range (IQR) for non-normally distributed variables and means and standards deviations for normally distributed variables. We computed overall scores of the vasomotor and sexual domains as well as the newly developed psychosocial domain and the entire MENQOL scale. Because some items from the physical domain were added to the psychological domain, we could not include the physical domain in the analysis—however, we used overall scores of the MENQOL scale (which comprises physical symptoms as well as other menopausal symptoms) in the analysis associated with ATMS.

Spearman’s rho correlations were performed to identify variables that are associated with the outcome variables. Hypotheses 1–5 proposed that vasomotor symptoms and weight gain contribute to symptoms of anxiety, depression, psychological distress, and memory problems directly and indirectly through fatigue and difficulty sleeping. To test these hypotheses, we examined four path models in Amos statistical software using maximum likelihood estimation. Outcome variables were depression, anxiety, psychological distress, and memory problems. Vasomotor symptoms and weight gain were used as predictors in all models. The latter is an item on the physical domain of the MENQOL scale, and it expresses the extent of being bothered by weight gain. Sleep loss/difficulty sleeping and fatigue were included as mediators (Figure 1). A bootstrap with 2000 iterations was used to obtain 95% bias-corrected confidence interval for all effects. Standard indices used to assess model-data fit are the chi-square statistic, comparative fit index (CFI), Tucker—Lewis index (TLI) (CFI and TLI should be >0.90 for an acceptable fit or greater for a good fit), and the root mean square error of approximation (RMSEA, which should be <0.08) [27,45]. Insignificant paths were trimmed in order to improve model fit. All path coefficients and correlations are expressed as standardized estimates.

To achieve the second aim, we conducted a multiple linear regression model involving only variables that had strong significant correlations with ATMS. Because of the small sample size, we limited the number of independent variables incorporated in the regression model: predictors were inserted in the model using backward method to allow removal of insignificant variables. All analyses were conducted in SPSS version 22 (IBM, USA) and AMOS 24 (IBM, USA). Significance was considered at a probability of 0.05 based on a two-tailed test.

## 3. Results

Participant women (mean age = 54.88 ± 6 years) were mostly married (71.2%), while 10.2% were single or widowed and 8.5% were divorced. Only 1.7% graduated the university, 8.3% were illiterate, and the rest of the participants (16.7%, 21.7%, and 26.7%) had elementary school, junior high school, and high school education, respectively. Most women were unemployed (83.4%). Only 6.7% were smokers. Around half the participants never used (45%) or used (55%) oral contraceptive pills. The majority of women were postmenopausal (86.7%), while only 13.3% were perimenopausal. The average time elapsed since the last menstrual period was 6.13 ± 5.22 years. Only 10% reported lifetime use of HRT, and 5% were current HRT users. Most participants (61.7%) rated their health condition as good, while 20% rated it as very good, and 18.3% rated it as poor.

Table 1 shows the percentage of participants encountering psychological symptoms and the extent to which they are bothered by these symptoms. Fatigue expressed as “feeling tired or worn out” and “feeling a lack of energy” was the most experienced (65.0%) and the most bothersome symptom (median = 4, IQR = 5). Difficulty sleeping was the second most frequent (61.7%) and the second most bothersome symptom (median = 4, IQR = 5) followed by “feeling anxious or nervous” (58.3%), “experiencing poor memory” (55.0%), and “feeling depressed, down or blue” (50.0%). 

Weight gain was experienced by 63.3% of the participants, and the degree of being bothered by this symptom in the whole sample was moderate (3.87 ± 2.48). This variable was involved in path analysis and regression models as a predictor.

Most psychological variables addressed in this study (variables 1 to 6 in Table 2) demonstrated significant positive correlations with vasomotor symptoms, sexual symptoms, weight gain, and total MENQOL scores. We found no associations between scores of all psychological variables with any sociodemographic and clinical characteristics of the participants such as age, level of education, marital status, menopausal status, smoking, use of HRT, occupation, health status, and age of onset of menopause (Supplementary Materials). 

To test hypotheses 1 to 5, four path models were conducted to examine (a) direct associations of vasomotor symptoms, weight gain, difficulty sleeping, fatigue, with symptoms of anxiety, depression, psychological distress, and memory problems; and (b) indirect associations of vasomotor symptoms and weight gain with anxiety, depression, psychological distress, and memory problems through difficulty sleeping and fatigue. Path models comprising difficulty sleeping were not significant, and thus were trimmed to improve model fit. Table 3 shows that the four trimmed path analysis models, with effects of vasomotor symptoms and weight gain mediated by fatigue, had acceptable fit on all measures, albeit RMSEA was a bit high in models predicting anxiety and psychological distress (0.090 and 0.096, respectively). The full path models accounted for 47.6%, 44.5%, 56.6%, and 29.1% of the variances in anxiety, depression, psychological distress, and memory problems, respectively.

**Figure 3:**
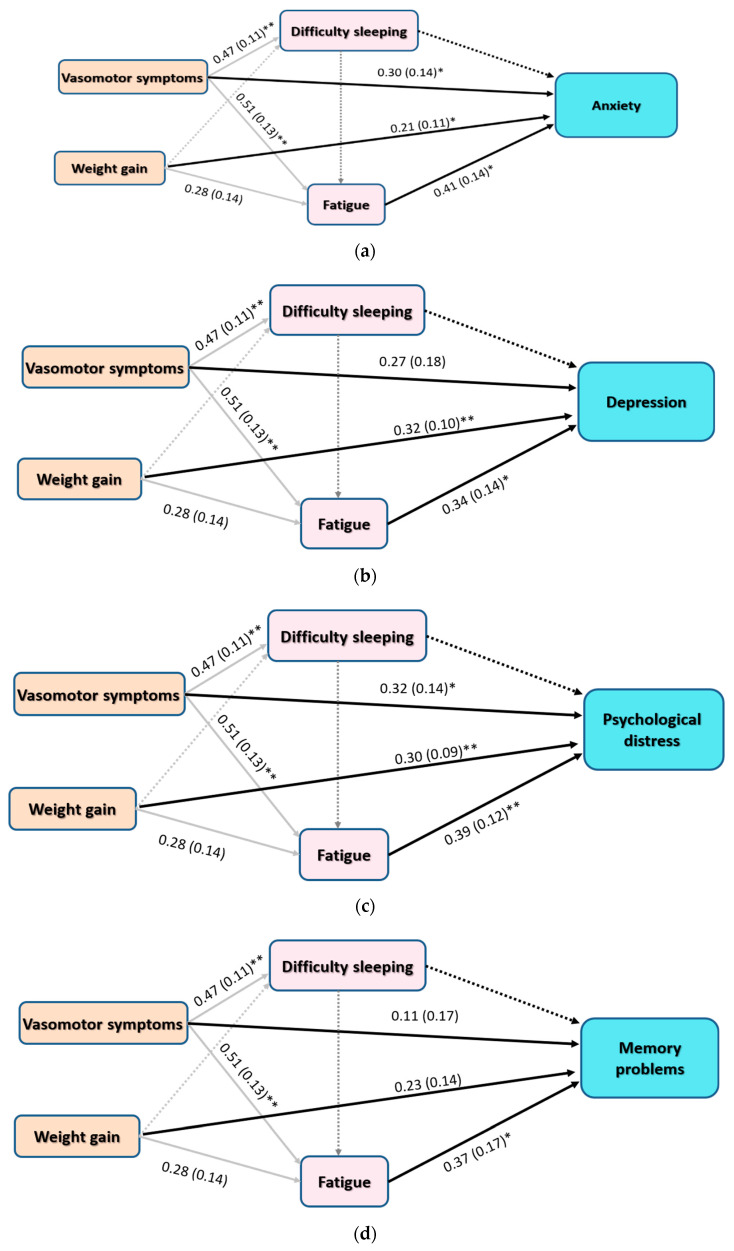
Results of trimmed path analysis models with standardized beta weights, standard errors, and significance values depicting relationships of vasomotor symptoms, weight gain, difficulty sleeping, and fatigue with symptoms of anxiety (Panel **a**), depression (Panel **b**), psychological distress (Panel **c**), and memory problems (Panel **d**) among menopausal women. * *p* < 0.05, ** *p* < 0.01. Dotted lines represent trimmed paths.

In all models, as shown in Figure 3, there was a statistically significant path coefficient for the direct effect of vasomotor symptoms on fatigue (β = 0.51, *p* = 0.001) while the effect of weight gain on fatigue approached statistical significance (β = 0.28, *p* = 0.05)—the full model accounted for 34.4% of the variance in fatigue (*p* = 0.009, 95% CI: 0.167 to 0.478). Only vasomotor symptoms were significantly associated with difficulty sleeping (β = 0.47, *p*= 0.001), and the full model accounted for 21.9% of the variance in difficulty sleeping (*p* = 0.001, 95% CI: 0.115 to 0.425). 

According to Figure 3a, there were statistically significant path coefficients (*p* < 0.05) for the direct effects of vasomotor symptoms, weight gain, and fatigue on anxiety symptoms (β = 0.21, 0.30, and 0.41, respectively). Both vasomotor symptoms and weight gain had significant indirect effects on anxiety through fatigue (β = 0.15 and 0.19, *p* = 0.008 and 0.042, respectively). Figure 3b shows a direct positive effect of weight gain on depression (β = 0.32, *p* = 0.006) along with a significant indirect effect of vasomotor symptoms through fatigue (β = 0.22, *p* = 0.011) while the indirect effect of weight gain through fatigue tended to be significant (β = 0.28, *p* = 0.050). According to Figure 3c, vasomotor symptoms, weight gain, and fatigue had positive direct effects on psychological distress (β = 0.32, 0.30, and 0.39; *p* = 0.018, 0.002, and 0.005, respectively). Fatigue significantly mediated the relationship of vasomotor symptoms and weight gain with psychological distress (β = 0.54 and 0.70, *p* = 0.002, and 0.045). Only fatigue expressed a significant direct effect on memory problems (β = 0.37, *p* = 0.042) (Figure 3d). It also significantly mediated the effect of vasomotor symptoms and weight gain (β = 0.07 and 0.09, *p* < 0.05). Please check Supplementary Materials for further details.

Women taking part in this study had an average attitude score of 2.45 ± 0.27 (min = 1.71, max = 3.12). According to Table 4, more than two thirds of the participants (71.7%) agreed or strongly agreed that “women should expect some trouble during menopause” while 76.7% viewed “menopause as one of the biggest changes that happen in a woman’s life” and agreed that “a woman should see a doctor at menopause”; these two attitudes were the most negative ones averaging 1.98 and 1.95, respectively. More than half of the participants (61.7%) agreed or strongly agreed that menopausal women feel freer to do things for themselves. Most participants (around 75%) disagreed that most women become disagreeable during menopause, menopausal women do not consider themselves real women, and that they consider menopause as an excuse for getting attention. The least endorsed attitude (15%) was that menopausal women are more interested in sex than they were before.

No associations were found between ATMS and sociodemographic and clinical characteristics of the participants (Supplementary Materials). ATMS expressed significant negative correlations with most menopausal symptoms—correlations with difficulty sleeping and sexual symptoms were not significant (Table 2). Accordingly, a multiple regression model was run to check if symptoms strongly correlated (*p* < 0.001) with ATMS (anxiety and psychological distress, vasomotor symptoms, weight gain, and total MENQOL scores) may predict attitudes toward menopause. Among different menopausal symptoms, only vasomotor symptoms and weight gain could explain 16.1% of the variance in attitudes toward menopause. However, only vasomotor symptoms expressed a significant effect (β = −323, *p* = 0.011, CI: −0.026 to −0.003, Supplementary Materials).

## 4. Discussion

This study investigated the hypotheses that high levels of vasomotor symptoms and increased body weight would be associated with high sense of fatigue, sleep loss, anxiety, and depressive symptoms, as well as overall psychological distress and memory problems. The findings support these hypotheses. In other words, the multiplicity and severity of menopausal symptoms may significantly endanger emotional and cognitive wellbeing in midlife women. The study also examined the relationship between the severity of menopausal symptoms and attitudes toward menopause. As expected, overall scores of the MENQOL scale as well as scores of its subdomains were negatively correlated with ATMS scores, indicating higher negative attitudes toward menopause among women with more severe symptoms. However, vasomotor symptoms seem to be the most influential in shaping attitudes toward menopause.

In this sample, vasomotor symptoms had significant effects on symptoms of anxiety and overall psychological distress both directly and indirectly through fatigue. On the other hand, vasomotor symptoms had no direct effect on symptoms of depression, despite the strong correlation noticed between these symptoms. Such absence of association may be due to the fact that this study used rather rough measures to evaluate depressive symptoms. Incorporation of more objective outcome indicators will certainly improve the quality of similar future studies. Nonetheless, fatigue significantly mediated the relation between vasomotor symptoms and most psychological climacteric symptoms, including depressive symptoms and memory problems (Figure 3). In other words, women with more severe vasomotor symptoms would experience more severe symptoms of fatigue, anxiety, depression, psychological distress, and cognitive alterations; the opposite is true. These finding are consistent with those reported in other studies. In this regard, Elavsky and Gold (2009) reported a positive direct effect of hot flashes on depressed mood and fatigue among menopausal women [27]. In accordance with our results, higher scores on the Menopause Rating Scale significantly predicted higher scores on the Hamilton Depression Rating Scale among menopausal women in Ecuador [47]. Cumulative knowledge supports the link between menopausal symptoms (particularly, vasomotor symptoms) and the occurrence of anxiety and depressive disorders. In this respect, a former systematic review identified a relation between anxiety symptoms and vasomotor symptoms in nine studies; two of them reported the prevalence of panic disorder [48]. Another review reported that vasomotor symptoms, vaginal dryness, and dyspareunia significantly correlate with anxiety and depressive symptoms. Poor sleep quality associated with hot flashes and night sweat also contributes to anxiety and depressive symptoms [16]. Nevertheless, difficulty sleeping, which strongly correlated with vasomotor symptoms in our study, had no effect on all psychological and memory outcomes. Again, this might be related to the fact that using one item on the MENQOL scale is not sufficient to reflect on all aspects of sleep quality. 

Studies examining the role of fatigue in the relationship between vasomotor symptoms and mood dysregulation in menopausal women are quite scarce. In line with our findings, a single study reported a direct effect of hot flashes on depressed mood and fatigue, along with a moderate association between depressed mood and fatigue. Both symptoms heightened perceived stress among American peri- and postmenopausal women [27]. Studies investigating conditions with alterations in feminine hormones demonstrate vivid relationships between fatigue and depression. In one study, breast cancer patients with high cognitive fatigue exhibited higher depressive and menopausal symptoms compared with counterparts with low cognitive fatigue [49]. A recent meta-analysis reports a strong relationship between fatigue and depressive symptoms during the first few months following childbirth, which correspond to hormonal drop during the postpartum period [50]. Around 70% of patients with chronic fatigue also suffer from depressive disorders and other affective problems. Research views fatigue and mood dysregulations as co-associated disorders that represent clinical manifestations of shared neurological pathologies related to inflammation, oxidative/nitrosative stress, and abnormal activity of the hypothalamic–pituitary–adrenal axis [26]. On the other side, addressing vasomotor symptoms through hypnosis is reported to reduce current fatigue and improve sleep and quality of life in postmenopausal women [29]. Likewise, treating menopausal fatigue with armodafinil was associated with improvement in depressive symptoms, insomnia, cognitive function, and total scores of the MENQOL scale [23]. In the meantime, a current meta-analysis revealed that use of armodafinil as an adjunctive treatment for depressive and bipolar disorders improves responsiveness to antidepressants [51]. Altogether, fatigue experienced by peri- and postmenopausal women may alter psychological wellbeing, and proper management of fatigue may be essential to prevent the development of chronic fatigue syndrome and mood disorders. 

Weight gain was a strong positive predictor of both depressive and anxiety symptoms as well as total psychological distress among our participants. Additionaly, weight gain exerted significant indirect effects on symptoms of anxiety, distress, and memory problems through the mediating effects of fatigue. Consistent with our results, Elavsky and Gold (2009) reported a significant contribution of BMI to symptoms of fatigue, depressed mood, and perceived stress among postmenopausal women [27]. Altogether, these findings emphasize the biological connections reported in the literature between sex hormones, obesity, mood dysregulations, and cognitive performance in humans. Research denotes that the effect of estradiol (E2) is more important than that of testosterone on the signaling pathways involved in the development of insulin resistance in healthy, young postmenopausal women [52]. Insulin resistance is associated with mitochondrial dysfunction, high production of free radicals, and high production of inflammatory mediators. High fat mass, a main source of adipose tissue dysfunctions, promotes insulin resistance and systemic inflammation via activation of macrophages and toll-like receptor 4 [53,54]. Neuroinflammation is a key pathological feature in disorders involving mood dysregulation such as depression and anxiety [55], as well as in cognitive alterations, which occur within the spectrum of normal cognitive aging, and in neurodegenerative disorders such as Alzheimer’s disease [11,13,14,15]. In fact, obesity and metabolic syndrome—characterized mainly by dysregulated metabolism of lipids and glucose—are independently associated with depression, and together they synergize its inflammatory pathway and worsen depressive symptoms and cognitive functions [56,57]. Moreover, depression induced by a high-fat diet is associated with alterations in intestinal microbiota, intestinal neuropeptides, and brain metabolome; it is also resistant to imipramine treatment [35]. On the other side, neurotrophic factors involved in high neuroinflammatory states such as ciliary neurotrophic factor affect food intake and body weight in experimental models of obesity induced by high-fat diet through a mechanism that involves influencing the activity of neuronal circuits and pathways located in the arcuate nucleus of the hypothalamus [58]. The hypothalamus plays a major role in the production of gonadotropin-releasing hormone and sex steroids [59]. Our findings, along with this detailed biological overview, highlight the importance of monitoring changes in body weight among menopausal women and addressing obesity as a major risk factor for neuropsychiatric disorders such as depression, anxiety, and Alzheimer’s disease.

Data of this study displayed no associations between psychological symptoms and subject characteristics such as age, menopausal status, etc. Consistent with our results, Olofsson and colleagues [36] reported no association between menopausal status and menopausal symptoms (except for vasomotor symptoms and joint pain) among menopausal women in Sweden. Likewise, the intensity of menopausal symptoms among Iranian women correlated only with marital status and husbands’ level of education [60]. On the contrary, these findings are incongruent with those reported among Jordanian and Lebanese women [9,10] where the severity and occurrence of overall menopausal symptoms correlated with age, family income, level of education, parity, perceived health status, and menopausal status. This discrepancy may be related to the fact that most participants in the current study were postmenopausal, which implies minor differences in variables related to age and health status. In addition, the socioeconomic level in the UAE (an oil-rich and economically vivid country) is different from occupied countries like Lebanon and Jordan. In this respect, further investigations that compare differences between menopausal women in different Arab countries are necessary. 

Overall, attitude mean score in this sample represents mild to moderate positive attitudes toward menopause. Sociodemographic and clinical characteristics were not correlated with attitudes toward menopause. On the other hand, increases in the degree of being upset about encountering anxiety symptoms, vasomotor symptoms, distress symptoms, sexual symptoms, overall MENQOL symptoms, and weight gain (the latter may reflect dissatisfaction with body image) were associated with a reduction in attitude scores, i.e., a rise in negative attitudes. A number of studies report consistent findings. In this regard, aged Turkish women who experienced fewer depressive symptoms and more positive body image experienced more positive attitudes towards menopause compared with women with more depressive symptoms and body image disturbance [19]. Similarly, attitudes toward menopause in climacteric Iranian women were significantly affected by excessive night sweat, poor memory, and sleeplessness [60]. An existing systematic review reports that in ten out of thirteen studies, negative attitudes are associated with symptoms severity during menopause transition [61]. In our study, attitudes could not significantly predict any of the neuropsychiatric symptoms (Supplementary Materials). Altogether, these findings denote that experiencing intense menopausal symptom may promote the development of negative attitudes toward menopause.

## 5. Strengths and Limitations

Apart from confirming the main role of vasomotor symptoms in psychological distress, this study also shows that fatigue and changes in body composition that follow estrogen drop during menopause, particularly weight gain, are key contributors to psychological distress among menopausal women. Crucial practical implications can be derived from these findings. In this context, various levels of responses may be adopted to improve quality of life in midlife women by addressing vasomotor symptoms, fatigue, and distress. At the level of healthcare systems and work organizations, prompt identification of women in need for specific psychological care is essential. It is critical that appropriate care should be delivered in a sensitive and non-stigmatizing manner. Organizational support should be properly tailored to promote the development of social support networks and provide interventions that address matters of fatigue, stress, exhaustion, and burnout among working menopausal women [4,22]. Such interventions may include mindfulness classes and well-designed physical activity. The latter is a common effective treatment of depressive/anxiety disorders and fatigue [26], and it is reported to abolish vasomotor symptoms in menopausal women. However, the responsiveness of women with high fat mass is rather limited [27]. Hence, weight control measures such as diurnal intermittent fasting and dietary interventions (e.g., caloric restriction, high fiber diet, and herbal supplements) may be beneficial [3,62]. Reports from studies employing hypnosis and armodafinil in menopausal women uncovered multidimensional effects, including improvements in fatigue, sleep, emotional response, and quality of life [23,29]. On the personal level, it might be important that menopausal women receive structured education about the nature of menopause [4,10]. Cognitive behavioral interventions, similar to those used for patients suffering from depression or anxiety, may be helpful—for example, helping patients learn and practice monitoring changes in their mood, sleep, cognition, and vasomotor symptoms in response to stressful events, social interactions, food intake and the like. This strategy, from a theoretical point of view, may help these women restructure their personal resources to promote better coping and develop higher levels of resilience. However, research is needed to evaluate the effectiveness of self-monitoring and self-care strategies among midlife women. 

Despite the insights provided by the current study, it has a number of limitations, which may limit generalizability of the findings. First, the sample size is relatively small, although a power analysis was estimated before data collection [4]. Based on a previous study reporting a strong correlation between scores of the MENQOL scale and BDI [42], variables reflecting on symptoms of fatigue, sleep loss, memory problems, depression, and anxiety were derived from the MENQOL scale instead of being estimated by specific measures (e.g., Mini-Mental State Examination or BDI), which may have resulted in measurement errors. Furthermore, it was not possible to test the validity (e.g., dimensionality and factorial purity) of the MENQOL and ATMS because of the small sample size, albeit results of reliability testing indicate good internal consistency of both scales. In addition, weight gain was subjectively reported by the participants and not objectively measured, which may infer some degree of self-reported bias (e.g., body image disturbance) rather than actual fatness. Lack of assessment of psychological symptoms before the onset of menopause as well as using a cross-sectional design cast doubt on the direction of the hypothesized relationships in the current study. In this sense, there is a possibility that women with higher levels of mood dysregulation develop chronic fatigue, which increases bodily distress by increasing the release of cytokines and free radical, resulting in more severe vasomotor symptoms. Therefore, it is necessary that future studies test the plausibility of the causal pathways involving vasomotor symptoms, fatigue, and emotional distress. This study did not address other important alternative causal pathways such as social support, physical activity, coping style, and levels of resilience—which may also need further exploration in future investigations.

## 6. Conclusions

Vasomotor symptoms and weight gain contribute to fatigue, mood dysregulation, sleep disturbance, memory problems, and overall distress among menopausal women. The relationship between these variables is either direct or mediated by fatigue. Most menopausal symptoms correlate with negative attitudes toward menopause; however, only vasomotor symptoms could predict negative attitudes toward menopause. Overall, these findings signify a widespread of psychological distress among menopausal women and highlight a key role of vasomotor symptoms, fatigue, and obesity in the development of psychological distress and negative attitudes toward menopause (only vasomotor symptoms). Hence, special measures directed toward mitigation of fatigue and alleviation of psychological distress may be beneficial.

## Figures and Tables

**Table 1:** Prevalence of psychological symptoms among the study participants.

Symptoms	With Experience NO (%)	Without Experience NO (%)	Degree of Being Bothered by Symptoms Median (IQR)
Being dissatisfied with my personal life	16 (26.7%)	44 (73.3%)	1 (2)
Feeling anxious or nervous	35 (58.3%)	25 (41.7%)	3 (5)
Experiencing poor memory	33 (55.0%)	27 (45.0%)	3 (4)
Accomplishing less than I used to	25 (41.7%)	35 (58.3%)	1 (3)
Feeling depressed, down or blue	30 (50.0%)	30 (50.0%)	2 (4)
Being impatient with other people	28 (46.7%)	32 (53.3%)	1 (4)
Feelings of wanting to be alone	21 (35.0%)	39 (65.0%)	1 (3)
Feeling tired or worn out	39 (65.0%)	21 (35.0%)	4 (5)
Difficulty sleeping	37 (61.7%)	23 (38.3%)	4 (5)
Decrease in stamina	29 (48.3%)	31 (51.7%)	1 (4)
Feeling a lack of energy	39 (65.0%)	21 (35.0%)	4 (5)
Fatigue▲	Minimum = 2	Minimum = 16	7.5 (9)
Anxiety▲	Minimum = 2	Minimum = 16	5.5 (8)
Depression▲	Minimum = 4	Minimum = 11	7 (11)
Psychological distress▲	Minimum= 9	Maximum= 69	21.5 (23)

▲: Variables are computed according to Figure 2.

**Table 2:** Correlations between psychological variables and attitudes toward menopause with different menopausal symptoms.

Variables	1	2	3	4	5	6	7	8	9	10
1. Anxiety	--									
2. Depression	0.793 **	--								
3. Psychological distress	0.893 **	0.861 **	--							
4. Memory problems	0.537 **	0.517 **	0.654 **	--						
5. Difficulty sleeping	0.214	0.242	0.457 **	0.177	--					
6. Fatigue	0.593 **	0.491 **	0.664 **	0.510 **	0.361 **	--				
7. Vasomotor symptoms	0.540 **	0.486 **	0.607 **	0.370 **	0.449 **	0.540 **	--			
8. Sexual symptoms	0.426 **	0.427 **	0.443 **	0.087	0.260 *	0.475 **	0.435 **	--		
9. Total MENQOL	0.823 **	0.738 **	0.898 **	0.582 **	0.411 **	0.826 **	0.681 **	0.628 **	--	
10. ATMS	−0.374 **	−0.309 *	−0.390 **	−0.268 *	−0.191	−0.304 *	−0.358 **	−0.201	−0.334 **	--
11. Weight gain▲	0.427 **	0.437 **	0.476 **	0.372 **	0.121	0.372 **	0.225	0.368 **	0.499 **	−0.350 **

* *p* < 0.05, ** *p* < 0.01, MENQOL: Menopause-Specific Quality of Life scale, ATMS: attitudes toward menopause scale, ▲: Weight gain is expressed as the degree of being bothered by increased body weight; it ranged from 1 (no weight gain) to 8 (being extremely bothered by weight gain).

**Table 3:** Summary of model-data fit and the percentages of variance explained by trimmed path models.

Outcome Variables	χ^2^	P	CFI	TLI	RMSEA	R^2^	SE	P	95% CI
Anxiety	5.932	0.204	0.976	0.941	0.090	0.476	0.088	0.009	0.298 to 0.591
Depression	4.203	0.379	0.997	0.993	0.029	0.445	0.090	0.013	0.258 to 0.557
Psychological distress	6.180	0.186	0.977	0.942	0.096	0.566	0.080	0.008	0.406 to 0.671
Memory problems	4.731	0.316	0.988	0.970	0.056	0.291	0.102	0.013	0.115 to 0.425

N.B. Given that sexual symptoms had strong positive correlations with symptoms of anxiety, depression, and psychological distress (Table 2), they were included in initial models as a predictor of these symptoms. However, these models exhibited poor fit, even after trimming insignificant paths. Associations involving sexual symptoms were less significant (all *p* values < 0.05) compared with those involving vasomotor symptoms and weight gain (*p* values ranged from <0.05 to <001). Therefore, sexual symptoms were removed from all models, which resulted in good model-data fit.

**Table 4:** The percentage of participants who agreed and strongly agreed to items of the attitudes toward menopause scale, along with mean ± SD for each item.

	Items	Agreement (%)	Mean ± SD
1	A woman should see a doctor at menopause	46 (76.7%)	1.95 ± 0.84
2	Menopause is one of the biggest changes that happens in a woman’s life	46 (76.7%)	1.98 ± 0.82
3	A woman is concerned about how her husband will feel about her after menopause	35 (58.3%)	2.14 ± 0.85
4	Menopause is an unpleasant experience	37 (61.7%)	2.19 ± 0.82
5	After the change of life, a woman feels freer to do things for herself	37 (61.7%)	2.80 ± 0.71
6	Women generally feel better after menopause	29 (48.3%)	2.52 ± 0.71
7	Women are generally calmer and happier after the change of life	28 (46.7%)	2.50 ± 0.71
8	A woman has a broader outlook on life after the change	28 (46.7%)	2.61 ± 0.74
9	Menopause is a disturbing thing that women naturally dread	36 (60%)	2.21 ± 0.81
10	Women should expect some trouble during menopause	43 (71.7%)	2.19 ± 0.71
11	A woman’s body may change in menopause, but otherwise, she doesn’t change much	35 (58.3%)	2.61 ± 0.83
12	It is no wonder women feel down in the dumps at the time of menopause	31 (51.7%)	2.39 ± 0.79
13	Life is more interesting for a woman after menopause	24 (40%)	2.47 ± 0.80
14	Changes inside the body that women cannot control cause all the trouble during menopause	33 (55%)	2.37 ± 0.72
15	A woman gains more confidence in herself after the change of life	23 (38.3%)	2.39 ± 0.77
16	Going through menopause really does not change a woman in any important way	21 (35%)	2.29 ± 0.77
17	Women worry about losing their minds during menopause	23 (38.3%)	2.54 ± 0.85
18	Women think of menopause as the beginning of the end	24 (40%)	2.55 ± 0.86
19	The only difference between a woman who has been through menopause and one who has not is that one menstruates and the other doesn’t	30 (50%)	2.54± 0.89
20	In truth, just about every woman is depressed about menopause	40 (66.7%)	2.24± 0.75
21	Women often use the change of life as an excuse for getting attention	14 (23.3%)	2.74 ± 0.66
22	After the change of life, women do not consider themselves real women	14 (23.3%)	2.75 ± 0.76
23	It’s not surprising that most women become disagreeable during menopause	15 (25%)	2.78 ± 0.77
24	After the change of life, a woman has a better relationship with her husband	23 (38.3%)	2.34 ± 0.79
25	Many women think menopause is the best thing that ever happened to them	26 (43.3%)	2.50 ± 0.84
26	After the change of life, a woman becomes more interested in community affairs than before	40 (66.7%)	2.71 ± 0.70
27	Women who have trouble with menopause are usually those who have nothing to do with their time	33 (55%)	2.60 ± 0.72
28	Women who have trouble with menopause are those who are expecting it	26 (43.3%)	2.36 ± 0.74
29	Women often become self-centered at the time of menopause	34 (56.7%)	2.34 ± 0.78
30	A woman in menopause is apt to do crazy things she herself does not understand	18 (30%)	2.79 ± 0.73
31	Menopause is a mysterious thing that most women don’t understand	25 (41.7%)	2.54 ± 0.86
32	After menopause, a woman is more interested in sex than she was before	9 (15%)	2.02 ± 0.60
33	Unmarried women have a harder time than married women do at the time of menopause	22 (36.7%)	2.50 ± 0.84
34	A good thing about menopause is that a woman can quit worrying about becoming pregnant	38 (63.3%)	2.75 ± 0.82

## Supplementary Materials

The following are available online at https://www.mdpi.com/1660-4601/17/14/5028/s1, Factor analysis of the MENQOL scale, Correlations between psychological variables and ATMS with sociodemographic and clinical characteristics, Regression model predicting ATMS, and Overall summary of the output of path analysis.

## Abbreviations

5-HT2A	5-hydroxytryptamine
ATMS	Attitudes toward menopause scale
BDI	Beck depression inventory
ERs	Estrogen receptors
HRT	Hormonal replacement therapy
IQR	Interquartile range
MENQOL	Menopause-Specific Quality of Life
UAE	United Arab Emirates
